# Angiotensin receptors modulate the renal hemodynamic effects of nitric oxide in conscious newborn lambs

**DOI:** 10.14814/phy2.12027

**Published:** 2014-05-28

**Authors:** Angela E. Vinturache, Francine G. Smith

**Affiliations:** 1 The Alberta Children's Hospital Research Institute for Child and Maternal Health, University of Calgary, Calgary, Alberta, Canada; 2 The Department of Physiology & Pharmacology, University of Calgary, Calgary, Alberta, Canada

**Keywords:** AT1R, AT2R, cardiovascular, newborn, nitric oxide, physiology, postnatal

## Abstract

This study aimed to elucidate the roles of both angiotensin II (ANG II) receptors – type 1 (AT1Rs) and type 2 (AT2Rs) – separately and together in influencing hemodynamic effects of endogenously produced nitric oxide (NO) during postnatal development. In conscious, chronically instrumented lambs aged ~1 week (8 ± 1 days, *N* = 8) and ~6 weeks (41 ± 2 days, *N* = 8), systolic, diastolic, and mean arterial pressure (SAP, DAP, MAP) and venous pressure (MVP), renal blood flow (RBF), and renal vascular resistance (RVR) were measured in response to the l‐arginine analog, l‐NAME after pretreatment with either the AT1R antagonist, ZD 7155, the AT2R antagonist, PD 123319, or both antagonists. The increase in SAP, DAP, and MAP by l‐NAME was not altered by either ATR antagonist in either age group. The increase in RBF after l‐NAME was, however, altered by both ATR antagonists in an age‐dependent manner, which was mediated predominantly through AT2Rs in newborn lambs. These findings reveal that there is an age‐dependent interaction between the renin–angiotensin (RAS) and the NO pathway in regulating renal but not systemic hemodynamics through both ATRs, whereas AT2Rs appear to be important in the renal hemodynamic effects of NO early in life.

## Abstract (precis)

This study aimed to elucidate the roles of both angiotensin II receptors – type 1 (AT1Rs) and type 2 (AT2Rs) – separately and together in influencing hemodynamic effects of endogenously produced nitric oxide (NO) during postnatal development. The data generated provide evidence to support an age‐dependent interaction between the renin–angiotensin system and the NO pathway in regulating cardiovascular homeostasis early in life.

## Introduction

The renin–angiotensin (RAS) and nitric oxide (NO) systems are of primary importance in maintaining renal and cardiovascular homeostasis. Both systems function during fetal life and are further activated, both systemically and intrarenally, at birth. For instance, during the perinatal period, all components of the RAS including the primary effector peptide, angiotensin II (ANG II), are elevated (Broughton Pipkin et al. 1974; Siegel and Fisher 1980; Pelayo et al. 1981; Varga et al. 1981; Wilson et al. 1981; Monument and Smith 2003; Velaphi et al. 2007) and the NO pathway is activated (Ballevre et al. 1997; Tsukahara et al. 1997a,b; Solhaug et al. 2000; Sener and Smith 2001a,b, 2002). The physiological relevance of this is not known, although both systems may contribute to the adaptations to postnatal life.

An increasing body of evidence from human and animal studies shows that a delicate balance exists between the two vasoactive factors, NO and ANG II, in maintaining cardiovascular homeostasis, that involves multiple and complex pathways. For example, in adult mammals, ANG II stimulation of NO release appears to be mediated through activation of both AT1Rs and AT2Rs, yet their specific roles in this response have not been characterized. As reviewed by Toda et al. (2007), it is believed that NO‐mediated vasodilatation induced by AT1R inhibition may elicit an increase in NO availability and release through endothelial‐derived NOS. It has also been suggested that ANG II‐induced vasodilation mediated by NO release is a result of the predominant activation of AT2Rs and, subsequently, the bradykinin/NO/cGMP vasodilatory cascade (Carey et al. 2000; de Gasparo 2002; Bergaya et al. 2004; Yayama et al. 2006).

Much less is known about the balance between ANG II and NO in the newborn, and the specific roles for the ATRs, particularly AT2Rs, are largely unknown. Several in vitro studies have shown that the gene and protein expression of both ATRs and NOS isoforms undergo similar postnatal changes, and that ATRs are localized in the near proximity of the NOS isoforms. For example, within the preglomerular vasculature of the newborn piglet, AT1R gene expression is lowest at birth, and progressively increases during maturation, while AT2R mRNA and protein expression are highest at birth and decrease thereafter (Ratliff et al. 2009). We have also shown that AT2Rs are the predominant ATR subtype in the kidney of the newborn sheep and that intrarenal gene expression of both ATRs is altered developing ovine kidney in an age‐dependent manner (Vinturache et al. 2009). The renal distribution of NOS I and III also appears to be developmentally regulated, at least in some species; for example, renal NOS I (Solhaug et al. 2001) and NOS III mRNA (Rodebaugh et al. 2012) in piglets and adult pigs showed developmental patterns of expression. Fischer et al. (1995) measured the postnatal development of NOS I expression in the rat kidney and found that it follows a corticomedullary pattern ranging from single‐cell expression in the immature nephron to its full presence in the *macula densa* of nephrons in the mature kidney. Similarly for NOS III, Han et al. (2005) demonstrated a unique corticomedullary pattern in the newborn rat kidney which was distinct from that observed in the adult rat kidney. We have also shown that all three NOS isoforms are expressed in the cortex and medulla of the developing ovine kidney over the first 3 months of postnatal life. NOS I and NOS II mRNA levels were greater in cortex compared to medulla during the first 3 weeks, whereas NOS III mRNA levels were predominantly transcribed within the medulla. In all NOS isoforms, there was a decrease in cortical mRNA levels after 3–6 weeks of postnatal life (Davis et al. 2013).

To further elucidate the physiological relevance of the molecular expression alteration and the significance of the perinatal activation of the RAS and NO pathways, we employed pharmacological methods to study the roles of ANG II and NO in regulating cardiovascular homeostasis in conscious chronically instrumented lambs in the first week of life (newborn) and later in the postnatal period (~6 weeks). We explored the cardiovascular effects of the l‐arginine analog, l‐NAME (which inhibits endogenous NO production) (Sener and Smith 1999b, 2001a,b) as well as the selective AT1R antagonist, ZD 7155, and the AT2R antagonist, PD 123319 (Chappellaz and Smith 2005, 2007), and both antagonists together (Vinturache and Smith 2014) at different stages of postnatal maturation (~1 and 6 weeks). Administration of an AT1R antagonist alone was associated with a decrease in mean arterial pressure (MAP) and renal blood flow (RBF) and an increase in renal vascular resistance (RVR) in both age groups studied, with effects higher at 1 than 6 weeks (Chappellaz and Smith 2007; Vinturache and Smith 2014). An AT2R antagonist alone had no influence on systemic and renal hemodynamics at 1 or 6 weeks of postnatal life (Chappellaz and Smith 2007; Vinturache and Smith 2014), and did not alter the hemodynamic responses of the AT1Rs antagonists when both drugs were administered concomitantly (Vinturache and Smith 2014). We also found that hemodynamic responses to l‐NAME are considerably greater in the immediate newborn period as compared to later in life revealing a predominant role for endogenously produced NO as one of the major regulators of RVR in the newborn period. Systemic l‐NAME infusion produced a greater increase in MAP and RVR at 1 week as compared to 6‐week‐old lambs (Sener and Smith 1999b, 2001a, 2002). We also showed that plasma renin activity (PRA) decreased after l‐NAME, with a greater effect observed at 1 than 6 weeks (Sener and Smith 2002). This observation suggests that l‐NAME effects on renal hemodynamics may result, at least in part, from the decreased level of ANG II, evidence that advocates for an interaction between NO and RAS in early postnatal development.

More recently, we evaluated any potential interaction between AT1Rs and NO in modulating cardiovascular homeostasis and the arterial baroreflex control of heart rate in conscious lambs (Wehlage and Smith 2012). The combination of an AT1R antagonist and l‐NAME appeared to remove age‐dependent differences that we previously revealed in the arterial baroreflex control of heart rate during postnatal maturation (Sener and Smith 2001b; Monument and Smith 2003). This provided new evidence that there appears to be an important interaction between ANG II and NO in regulating cardiovascular homeostasis early in life, at least through activation of AT1Rs. Any role for AT2Rs in influencing the cardiovascular effects of NO early in life, or whether there is an interaction between AT1Rs and AT2Rs in regulating the hemodynamic responses to NO, is not yet known, forming the basis to our study.

Therefore, this study was designed to test the hypothesis (1) that AT2Rs mediate the modulatory effects of ANG II on hemodynamic effects of NO and (2) that AT2Rs influence the effects of AT1Rs on hemodynamic responses to NO, thereby further promoting an interaction between endogenously produced ANG II and NO in the newborn period, at a time when circulating levels of ANG II and NO are increased and AT2Rs predominate in the kidney and conduit arteries of sheep (Samyn et al. 1998; Cox and Rosenfeld 1999; Cox et al. 2005). To this end, the systemic and renal hemodynamic responses to an AT1R antagonist, an AT2R antagonist, and both antagonists combined, were evaluated before and after administration of l‐NAME, in conscious, lambs aged ~1 week – when circulating levels of ANG II and NO are increased and AT2Rs predominate in the kidney, heart, and conduit arteries of the sheep, and ~6 weeks – when circulating ANG II and NO levels as well as AT2R expression are decreased.

## Methods

### Animals

Experiments were carried out in two groups of conscious, chronically instrumented lambs aged ~1 week (newborn, *N* = 8) and ~6 weeks (older lambs, *N* = 8) (see Table 1 for demographic details). Animals were obtained from a local source (Woolfitt Acres, Olds, Alberta, Canada) and housed with their mothers in individual pens in the *vivarium* except during surgery and experiments. All surgical and experimental procedures were carried out in accordance with the “Guide to the Care and Use of Experimental Animals” provided by the Canadian Council on Animal Care and with the approval of the Animal Care Committee at the University of Calgary.

**Table 1.** Demographics and baseline measurements in conscious lambs

	1 Week	6 Weeks
*N*	8	8
Sex	2♀/6♂	6♀/2♂
Age (days)	8 ± 1	40 ± 3*
Body mass (kg)	7.6 ± 0.4	15.6 ± 3.3*
Kidney mass (g)	62.9 ± 15.1	80.8 ± 9.6*
MVP (mmHg)	2 ± 2	6 ± 2*
MAP (mmHg)	72 ± 2	79 ± 6*
SAP (mmHg)	103 ± 9	110 ± 6*
DAP (mmHg)	49 ± 6	53 ± 10*
HR (bpm)	202 ± 29	134 ± 7*
RBF (mL·g min^−1^)	1.8 ± 0.4	3.7 ± 1.1*
RVR (mmHg·mL·min^−1^·g^−1^)	39.4 ± 7.8	20.6 ± 7.1*

Data represent averages for the control periods from all three experiments. Values are mean ± SD. MAP, mean arterial pressure; SAP, systolic arterial pressure, DAP diastolic arterial pressure, MVP, mean venous pressure; HR, heart rate; RBF, renal blood flow; RVR, renal vascular resistance.

* *P* < 0.05 compared to 1 week.

### Surgical procedures

Using aseptic techniques and procedures previously described (Ebenezar et al. 2012; Vinturache and Smith 2014), surgeries were performed in newborn lambs 2–3 days after birth and in older lambs at least 4 days before the beginning of experiments. Briefly, after induction with a mask and 5% isoflurane in oxygen, anesthesia was maintained with isoflurane 1.5–3.0% in a 3:2 mixture of nitrous oxide and oxygen. Specially prepared polyethylene catheters (Tygon Microbore Tubing, Colorado Springs, CO) were inserted through femoral arteries and veins to the level of abdominal aorta and inferior vena cava, for recordings of central arterial and venous pressures and intravenous (i.v.) infusions of drugs and solutions during experiments. Through an incision in the right flank, the kidney was exposed and a precalibrated ultrasonic flow transducer (size 3–6S, Transonic Systems Inc., Ithaca, NY) was placed around the renal artery as previously described (de Wildt and Smith 1997) for later measurement of RBF. After incisions were closed, the catheters and the flow transducer cable were tunneled subcutaneously to exit the animal on the left and right flanks, respectively, and secured inside pouches of a body jacket (Lomir Inc., Montreal, Quebec, Canada). Lambs were allowed to recover from the effects of anesthesia and surgery in a critical care unit (Shor‐Line; Schroer Manufacturing Company, Kansas, MO) after which they were returned to the ewe. Antibiotics, Excenel^®^ (Ceftriofur, 2.2 mg/kg; Pfizer, Kirkland, Quebec, Canada), were administered intramuscularly prior to surgery and at 24 h intervals for the next 48 h. A minimum of 3 days was allowed for recovery from the effects of anesthesia and surgery, during which time the animals were trained daily for 1–2 h to rest comfortably in a supportive sling in the laboratory environment in which they were housed during the experiments.

### Experimental details

On the day of an experiment, the animals were removed from *vivarium* and placed in the supportive sling in the laboratory environment. A period of at least 60 min was allowed for hemodynamic equilibration during which time an i.v. infusion of 5% dextrose in 0.9% sodium chloride (4.17 mL·kg^−1^·h^−1^) was initiated and continued for the experiment using a microinfusion pump (Mi 60‐1B, World Precision Instruments Inc., Sarasota, FL) in order to maintain fluid and electrolyte balance. The left femoral venous and arterial catheters were connected to pressure transducers (Model P23XL; Statham, West Warwick, RI) to measure central venous and arterial pressures. The flow transducer placed around the renal artery was connected to a flow meter (T101; Transonics Systems Inc., New York, NY) for measurement of RBF. Hemodynamic variables were continuously recorded onto a polygraph (Model 7, Grass Instruments Inc., West Warwick, RI) and simultaneously digitized at 200 Hz using the data acquisition and analysis software package, PolyVIEW^™^ (Astro Med Inc., Grass Instrument Division, West Warwick, RI).

Three experiments were carried out in each lamb at intervals of 48 h to ensure adequate time for complete clearance of drugs between experiments (Chappellaz and Smith 2005, 2007). The three experiments were randomized to eliminate bias and were as follows:

Control + ZD 7155 + Vehicle + l‐NAME (experiment one)
Control + PD 123319 + Vehicle + l‐NAME (experiment two)
Control + ZD 7155 + PD 123319 + l‐NAME (experiment three)

Each experiment consisted of hemodynamic measurements for 30 min before (Control, C) and 30 min after i.v. administration of each ATR antagonist (ZD 7155 or PD 123319) or the combination of drugs (both ATRs antagonists together, ATRs antagonist and vehicle, and ATRs antagonist and l‐NAME).

ZD 7155 and PD 123319 were administered using a microprocessor‐controlled syringe pump (model 11, Harvard Apparatus, Holliston, MA). ZD 1755 and PD 123319 were administered as an i.v. bolus of 100 *μ*g·kg^−1^ followed by a constant infusion at 70 *μ*g·kg^−1^·h^−1^, whereas l‐NAME was administered i.v. as a bolus at a concentration of 20 mg·kg^−1^ as previously detailed (Wehlage and Smith 2012; Vinturache and Smith 2014), and vehicle was administered as an equivalent volume of saline.

### Drugs

ZD 7155 (5,7‐diethyL‐3,4‐dihydro‐1‐[[2′‐(1H‐tetrazoL‐5‐yl)[1,1′‐biphenyl]‐4‐yl]methyl]‐1,6‐naphthyridin‐2(1H)‐one hydrochloride; Tocris Bioscience, Ellisville, MO) is a potent and selective competitive antagonist for the AT1R (Junggren et al. 1996). ZD 7155 potency is ~10 times higher than that of losartan in antagonizing ANG II‐induced pressor effects in conscious normotensive and spontaneously hypertensive rats, yet unlike losartan, has no active metabolite which could impact on the physiological measurements (Junggren et al. 1996).

PD 123319 (1‐[[4‐(dimethylamino)‐3‐methylphenyl] methyl]‐5‐(diphenylacetyl)‐4, 5, 6, 7‐tetrahydro‐1*H*‐imidazo [4, 5‐c] pyridine‐6‐carboxylic acid ditrifluoroacetate; Tocris Bioscience, Ellisville, MO) is a potent, selective, reversible nonpeptide angiotensin AT2R receptor antagonist (Blankley et al. 1991).

The doses of ZD 1755 and PD 123319 were selected from dose–response studies conducted previously in our laboratory over the range of doses 0–1600 *μ*g/kg. The efficacy of inhibition of AT1Rs by ZD 7155 was assessed previously by testing the pressor response to i.v. administration of the half‐effective maximal concentration (EC_50_) of ANG II in carefully performed dose–response experiments in different age groups of lambs (Chappellaz and Smith 2005, 2007). In most studies involving the use of PD 123319 as a vasodilator, a dose similar to the one used in this study was shown to remain highly specific for AT2Rs, and sufficient to initiate a significant physiological effect (Ernsberger et al. 1992; Macari et al. 1993; Siragy and Carey 1996).

l‐NAME (*N*^*G*^‐nitro‐l‐arginine methyl ester hydrochloride, Sigma Aldrich Canada Ltd., Oakville, Ontario, Canada) is a member of a large class of l‐arginine analogs, which acts by competitively inhibiting l‐arginine binding with NOS. The dose used in these experiments was determined previously as that which inhibits intrarenal production of acetylcholine for 2 h (Sener and Smith 1999a,b, 2001a)

After experiments, animals were euthanized with pentobarbital sodium. Following post mortem inspection for verifying catheters and flow transducer placement, the zero offset of the flow transducer was recorded for correcting RBF. Both kidneys were removed, examined, and weighed to normalize RBF and RVR.

### Data handling and statistical analysis

All data were analyzed using PolyView^™^ software. Direct hemodynamic measurements (SAP, DAP, MAP, MVP, RBF) were averaged over consecutive 1, 10, and 30 min intervals. Heart rate (HR) was determined from the systolic peaks of the arterial pressure waveform. RVR was calculated as (MAP − MVP)/RBF, where MAP represents mean arterial pressure and MVP is mean venous pressure.

Data obtained from experiments were evaluated for statistical significance using ANOVA procedures for repeated measures with factors being age and treatment with application of Holm Sidak multiple comparison procedures where appropriate (IBM SPSS Statistics for Windows version 20.0; IBM Corp., Armonk, NY). Baseline values between the two age groups were tested using nonpaired Student's *t*‐tests. All data are expressed as mean ± SD. Significance was accepted at the 95% confidence interval. Data are graphically presented as a 30 min average for each treatment period.

## Results

There were significant differences in baseline hemodynamic variables between the two age groups. Arterial pressures (MAP, SAP, DAP), MVP, and RBF were higher, whereas RVR and HR were lower in lambs aged 6 weeks as compared to 1 week (Table 1). There were no significant differences between control values for any hemodynamic variables on the three separate experimental days in either age group.

### Systemic hemodynamic effects of l‐NAME: modulation by ATRs

**Figure 1.**
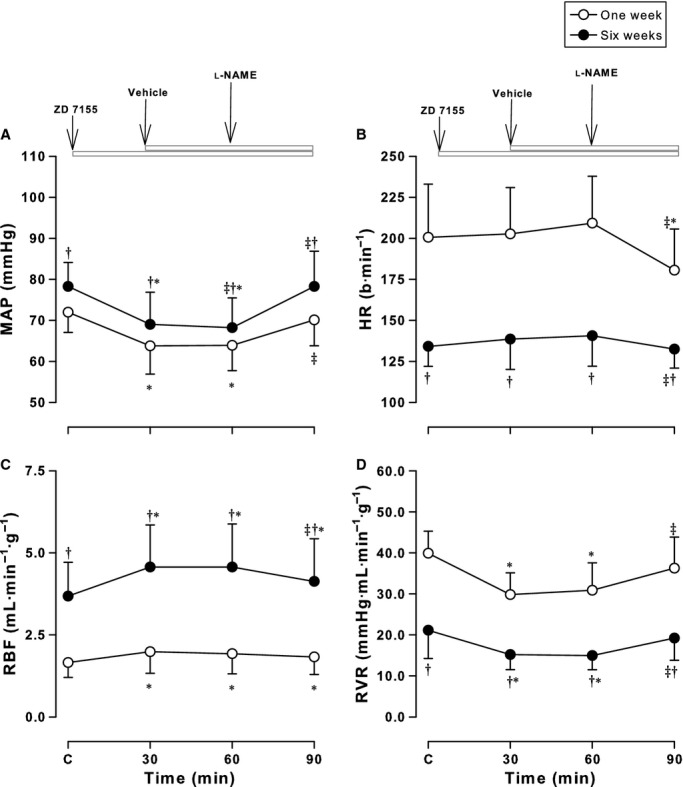
Effects of the AT1R antagonist, ZD 7155, on hemodynamic responses to l‐NAME. Mean arterial pressure (MAP) (A), heart rate (HR) (B), renal blood flow (RBF) (C), and renal vascular resistance (RVR) (D) measured before and after administration of the AT1R antagonist, ZD 7155 and l‐NAME in conscious lambs aged ~1 week (open symbols) and ~6 weeks (solid symbols). Values are mean ± SD. *P < 0.05 compared to C (Control); ^†^*P* < 0.05 compared to 1 week; ^&ddagger;^*P* < 0.05 compared to the previous treatment.

**Figure 2.**
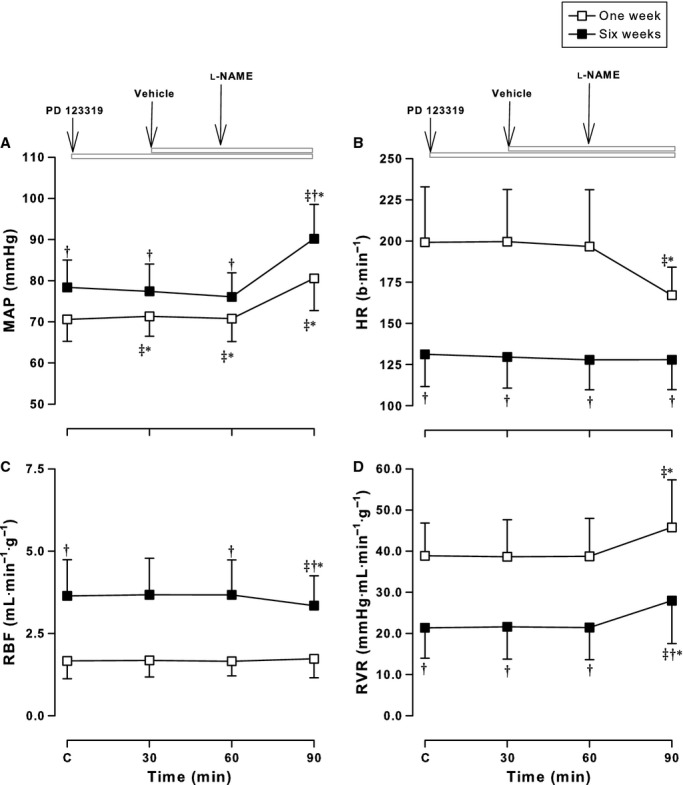
Effects of the AT2R antagonist, PD 123319, on haemodynamic responses to l‐NAME. Mean arterial pressure (MAP) (A), heart rate (HR) (B), renal blood flow (RBF) (C), and renal vascular resistance (RVR) (D) measured before and after administration of the AT2R antagonist, PD 123319 and l‐NAME in conscious lambs aged ~1 week (open symbols) and ~6 weeks (solid symbols). Values are mean ± SD. *P < 0.05 compared to C (Control); ^†^*P* < 0.05 compared to 1 week; ^&ddagger;^*P* < 0.05 compared to the previous treatment.

**Figure 3.**
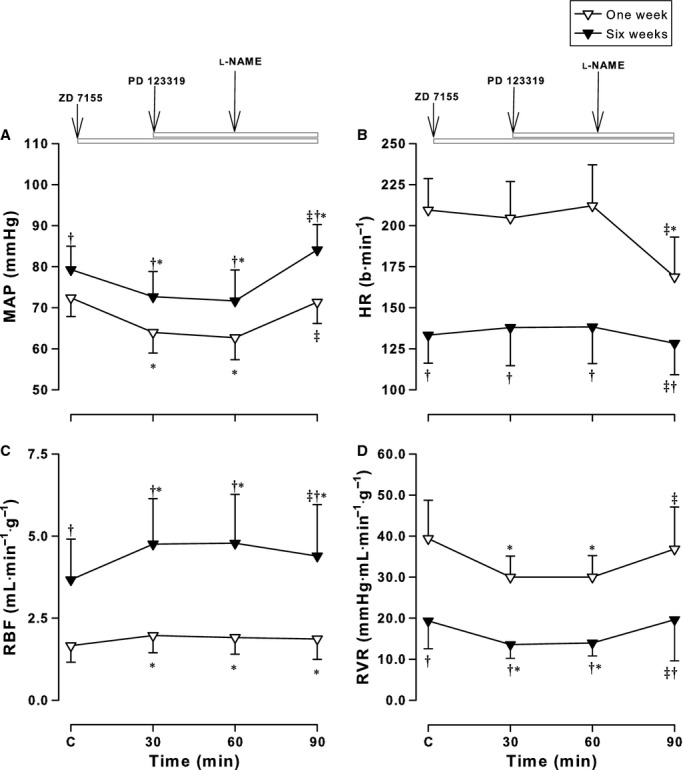
Effects of both the AT1 receptor antagonist, ZD 7155, and the AT2 receptor antagonist, PD 123319, on hemodynamic responses to l‐NAME. Mean arterial pressure (MAP) (A), heart rate (HR) (B), renal blood flow (RBF) (C) and renal vascular resistance (RVR) (D) measured before and after administration of ZD 7155 and PD 123319 and l‐NAME in conscious lambs aged ~1 week (open symbols) and ~6 weeks (solid symbols). Values are mean ± SD. *P < 0.05 compared to C (Control); ^†^*P* < 0.05 compared to 1 week; ^&ddagger;^*P* < 0.05 compared to the previous treatment.

Administration of NO synthesis inhibitor, l‐NAME was associated with a significant increase in MAP after all three combinations (from a significant effect of treatment, *F* = 431.4, *P* < 0.001). After the AT1R antagonist, ZD 7155 alone, MAP decreased in both groups (*P* < 0.001) as previously reported (Wehlage and Smith 2012; Vinturache and Smith 2014) (Fig. 1A). After l‐NAME, MAP increased to 70 ± 6 mmHg in newborns and 81 ± 10 mmHg in older lambs returning to control levels (Fig. 1A). The extent of this increase (~10–15 mmHg) is similar to the increase in MAP after l‐NAME alone as we have previously reported (Sener and Smith 2001a,b, 2002). Addition of the AT2R antagonist, PD 123319 did not influence this response in either age group (Fig. 3A). In contrast, MAP did not change after PD 123319 alone, but increased after addition of l‐NAME to 79 ± 5 mmHg in newborns and 91 ± 9 mmHg in older lambs, similar to the increase in MAP after l‐NAME alone reported previously (Sener and Smith 2001a,b, 2002) (Fig. 2A). The changes observed in MAP resulted from changes in both SAP and DAP in both groups of lambs (Table 2).

**Table 2.** Responses to l‐NAME: effects of ZD 7155, PD 123319, and both treatments

Age (weeks)	Variable		
	SAP (mmHg)	DAP (mmHg)	MVP (mmHg)
Experiment One			
Control			
1	103 ± 8	48 ± 6	2 ± 1
6	110 ± 11*	53 ± 7*	6 ± 2*
ZD 7155			
1	93 ± 11*	41 ± 6*	3 ± 2
6	101 ± 11**	45 ± 9**	5 ± 2*
Vehicle			
1	93 ± 9*	41 ± 6*	2 ± 2
6	101 ± 8**	45 ± 8**	6 ± 2*
l‐NAME			
1	103 ± 11*	48 ± 8*	2 ± 2
6	110 ± 10**	57 ± 10***	5 ± 3*
Experiment Two			
Control			
1	102 ± 10	49 ± 7	2 ± 1
6	109 ± 9*	52 ± 7*	6 ± 2*
PD 123319			
1	101 ± 10	49 ± 5	2 ± 1
6	111 ± 9*	51 ± 8*	6 ± 1*
Vehicle			
1	101 ± 9	48 ± 5	2 ± 1
6	111 ± 10*	51 ± 7*	6 ± 1*
l‐NAME			
1	112 ± 10**	57 ± 10**	3 ± 1
6	126 ± 14***	64 ± 10***	6 ± 1*
Experiment Three			
Control			
1	103 ± 8	49 ± 5	2 ± 1
6	109 ± 8*	53 ± 7*	7 ± 2*
ZD 7155			
1	93 ± 10 *	42 ± 5 *	2 ± 2
6	102 ± 8*	46 ± 4**	6 ± 2*
PD 123319			
1	91 ± 8 *	42 ± 6 *	2 ± 2
6	104 ± 13*	46 ± 4**	6 ± 2*
l‐NAME			
1	100 ± 10*	51 ± 7*	2 ± 2
6	117 ± 11***	60 ± 7***	5 ± 2*

Values are mean ± SD averaged over each 30 min measured before (Control) and 30 min after treatment with the AT1R antagonist, ZD 7155, and/or the AT2R antagonist, PD 123319, and 30 min after combined treatment with ZD 7155, PD 123319, or both plus l‐NAME. SAP, systolic arterial pressure; DAP, diastolic arterial pressure; MVP, mean venous pressure.

* *P* < 0.05 compared to Control.

* *P* < 0.05 compared to 1 week.

* *P* < 0.05 compared to ZD 7155 and PD 123319 treatment.

There were no effects of age or treatment on the MVP response to l‐NAME. MVP remained constant after all treatments in both groups of lambs (Table 2).

Heart rhythm responses to l‐NAME resulted from an overall effect of treatment (*F* = 60.8, *P* < 0.001), age (*F* = 579.5, *P* < 0.001) and an interaction between age and treatment (*F* = 10.0, *P* < 0.001). There were no changes in HR after ATR inhibition in either age group when administered separately or together, as previously reported (Vinturache and Smith 2014) (see Figs 1B, 2B, 3B). After l‐NAME addition to ZD 7155, PD 123319, or both antagonists, HR decreased in newborns by 20–35 bpm; this was similar to the decrease after l‐NAME alone (Sener and Smith 2001a,b, 2002). In contrast, addition of l‐NAME in older lambs did not significantly alter the HR. The l‐NAME responses in the present experiments were similar with the effects of l‐NAME alone on HR in 1‐ and 6‐week‐old conscious lambs previously reported by our group (Sener and Smith 2001a,b, 2002) (Figs 1–3).

### Renal hemodynamic responses to l‐NAME: modulation by ATRs

The panels in Figures 1–3 (C and D) illustrate the modulatory effects of ATRs on renal hemodynamic effects of l‐NAME. The RBF response to l‐NAME resulted from an effect of treatment (*F* = 161.0, *P* < 0.001), age (*F* = 639.2, *P* < 0.001), and an interaction between age and treatment (*F* = 94.7, *P* < 0.001).

Renal blood flow increased after ZD 7155 (Fig. 1C) but not PD 123319 (Fig. 2C) with greater responses in older lambs compared to newborns. As previously shown, the RBF responses to ZD 7155 were not altered by addition to PD 123319 in both groups of lambs (Vinturache and Smith 2014) (Fig. 3C).

In newborns, the RBF response to ZD 7155 or ZD 7155 plus PD 123319 was not altered by l‐NAME whereas in older lambs, l‐NAME attenuated by 12% the RBF response to ZD 7155 and ZD 7155 plus PD 123319 (Figs 1C, 3C). l‐NAME addition to PD 123319 in older lambs decreased RBF from 3.7 ± 1.1 to 3.3 ± 0.9 mL·min^−1^·g^−1^ (*P* < 0.001), while it did not alter the RBF in the newborns, in which RBF maintained in the range of 1.6–1.7 mL·min^−1^·g^−1^ for the duration of the experiment (Fig. 2C). Thus, the ~35% decrease in RBF in response to l‐NAME alone in newborn lambs previously reported by our group (Sener and Smith 2001a,b, 2002) was completely attenuated by PD 123319, and blunted by ZD 7155 in the present experiments. These observations suggest greater modulatory effects elicited by ATRs antagonists on the NO effects on blood flow to the kidney on the newborn period than later in the postnatal period.

Renal vascular resistance responses to l‐NAME were age dependent (*F* = 725.4, *P* < 0.001) and treatment dependent (*F* = 349.3, *P* < 0.001). As illustrated in Figure 1D, RVR responses to ATR inhibition were attenuated in both age groups by l‐NAME. RVR decreased from 39.9 ± 5.4 to 29.8 ± 5.3 mmHg·mL·min^−1^·g^−1^ in newborns and from 21.1 ± 6.9 to 15.2 ± 3.6 mmHg·mL·min^−1^·g^−1^ in older lambs after ZD 7155 (and similar to ZD 7155 plus PD 123319), as we have previously reported (Chappellaz and Smith 2007; Wehlage and Smith 2012; Vinturache and Smith 2014). These responses were attenuated by 6.4 mmHg·mL·min^−1^·g^−1^ in newborns and by 4.0 mmHg·mL·min^−1^·g^−1^ in older lambs (Fig. 1D) after l‐NAME. Although PD 123319 alone did not alter baseline RVR, addition of l‐NAME elicited a significant increase in RVR in both age groups by ~20% in newborns and ~30% in older lambs (Fig. 2D).

## Discussion

The present study was designed to investigate whether AT2Rs modulate the cardiovascular effects of endogenous ANG II through activation of AT1Rs in regulating hemodynamic responses to NO and whether these responses are developmentally regulated. To our knowledge, this is the first study to report on the role of AT2Rs in mediating the cardiovascular interactions between ANG II and NO in newborn animals under physiological conditions. Novel findings of this study are that (1) AT2Rs modulate renal but not systemic hemodynamic responses to l‐NAME; (2) there is no apparent interaction between AT1Rs and AT2Rs in modulating hemodynamic responses to endogenous NO. Therefore, we conclude that early in life, AT2Rs modify the effects of AT1Rs on renal but not systemic hemodynamic responses to l‐NAME. These findings provide additional evidence for a complex, developmentally regulated cross talk between ANG II and NO in regulating cardiovascular homeostasis during postnatal development.

Previously we explored the role of ATRs in the pressor and renal vascular responses to ANG II, by measuring the effects of the AT1R antagonist, ZD 7155, and the AT2R antagonist, PD 123319, in conscious developing lambs (Chappellaz and Smith 2005, 2007). Our results showed that the pressor and renal hemodynamic responses to ANG II were elicited through activation of AT1Rs but not AT2Rs. More recently, we explored the potential interaction between AT1Rs and AT2Rs in influencing hemodynamics in conscious developing lambs by measuring responses to both antagonists separately and together (Vinturache and Smith 2014). Our findings revealed that AT2Rs do not counterbalance the pressor and renal vasoconstrictor effects elicited by activation of AT1Rs in the immediate newborn period confirming our previous experiments (Chappellaz and Smith 2007; Vinturache and Smith 2014) and in contrast to that which occurs in adulthood (Carey et al. 2000; de Gasparo 2002; Bergaya et al. 2004; Yayama et al. 2006). Taken together, then, it appears that AT1Rs predominate in eliciting the hemodynamic effects of ANG II, whereas the role for the upregulated AT2Rs remains elusive (Qi et al. 2011), despite their preponderance in the systemic vasculature of the developing sheep and microswine (Cox and Rosenfeld 1999; Bagby and Angiotensin 2002; Cox et al. 2005).

We have also previously explored the cardiovascular effects of NO during postnatal development and showed that, in conscious lambs, hemodynamic effects of l‐NAME are age dependent, with greater systemic and renal hemodynamic responses elicited immediately after birth as compared to that seen later in life (Sener and Smith 1999a, 2001a). In addition, we showed that administration of l‐NAME was associated with a decrease in PRA which was greater in newborns compared to older animals (Sener and Smith 2002). Therefore, we reasoned that the age‐dependent hemodynamic effects of NO may be regulated through ANG II and mediated through activation of ATRs (Wehlage and Smith 2012). When assessing the arterial baroreflex control of HR, we demonstrated a decrease in HR after ZD 7155 alone as well as after l‐NAME at 1‐ but not 6 weeks and a decrease in the range over which the baroreflex operates after ZD 7155 as well as after ZD 7155 + l‐NAME (Wehlage and Smith 2012). These data revealed that, early in life, AT1Rs mediate the effects of ANG II on NO in regulating cardiovascular homeostasis and the arterial baroreflex control of heart rate, which may help to explain the activation of these two systems soon after birth (Wehlage and Smith 2012). Any role for AT2Rs alone, or in combination with AT1Rs, had not been explored. As an extension of these findings, the present experiments reveal that pretreatment with either the AT1R antagonist or the AT2R antagonist, alone or combined, does not attenuate the arterial pressure response to l‐NAME during postnatal maturation in conscious lambs. The increase in blood pressure observed after addition of l‐NAME was comparable to the responses to l‐NAME alone in conscious lambs of similar ages previously observed (Sener and Smith 1999b, 2001b; Wehlage and Smith 2012). Our findings therefore suggest that AT2Rs do not appear to influence the effects of AT1Rs on the blood pressure response to removal of endogenously produced NO early in life.

Solhaug et al. (1996a) showed in anesthetized piglets and adult pigs, that intrarenal infusion of l‐NAME after pretreatment with the AT1R antagonist A‐89291, also did not alter MAP. Baylis et al. (1993) and Sigmon et al. (1994) reported similar findings from anesthetized and conscious normotensive rats, respectively, thus supporting our observations in conscious lambs studied under physiological conditions. In the present study, the age‐dependent decrease in HR observed after ATR antagonists alone or combined with l‐NAME was similar to the effects of l‐NAME alone reported previously (Sener and Smith 2001a,b). In contrast, low‐dose l‐NAME infusion and systemic NO clamp in healthy volunteers augmented the systemic pressor response to ANG II and elicited a significant decrease in HR (Batenburg et al. 2004; van der Linde et al. 2005a), whereas in hypertensive subjects, unopposed activity of the RAS was not involved in the l‐NAME responses, similar to our present observations in conscious lambs (van der Linde et al. 2005b). In the latter study, as well as in young animals, the RAS system is activated, which could explain these conflicting differences.

Experiments in rats, swine, and pigs have revealed the intrarenal localization of ATRs in close proximity to NOS isoforms during development (Shanmugam and Sandberg 1996; Bagby et al. 2002; Han et al. 2005; Ratliff et al. 2009; Vinturache et al. 2009; Davis et al. 2013). As well, it appears that both ATRs and NOS enzymes undergo a corticomedullary regulation during maturation in a pattern distinct from the adult implicating their roles in perhaps regulating glomerular and/or tubular function. The role of AT2Rs in mediating the intrarenal interaction between ANG II and NO has been described for adult animals (Siragy and Carey 1997; Millatt et al. 1999). Such a relationship for the developing renal vasculature is not well understood, and any role for AT2Rs in influencing the renal effects of NO during development is not yet known. Therefore, we inquired on the facilitatory influence ATRs may exert on the functional maintenance of vascular tone by NO. In the present study, we observed an age‐dependent interaction between ANG II and NO mediated by AT1Rs in regulating the renal circulation, as previously reported by us. The fall in RBF observed after l‐NAME alone (Sener and Smith 1999b, 2001a; Wehlage and Smith 2012) was altered by pretreatment with ZD 7155 at 6 weeks and almost completely attenuated in newborns. However, the AT2R antagonist, PD 123319, attenuated the increase in RBF following l‐NAME at 1 week that we have previously observed (Sener and Smith 2001a; Wehlage and Smith 2012) providing new evidence for a critical role for AT2Rs in influencing the interaction between ANG II and NO soon after birth.

There were, however, no additional effects of PD 123319 combined with ZD 7155 on the renal hemodynamic responses to l‐NAME in developing lambs. Therefore, we report in this study for the first time that AT2Rs do not buffer the AT1R‐mediated hemodynamic effects of l‐NAME in the developing newborn kidney under physiologic conditions. Our research findings suggest that early in life there is either (1) a blunting effect on NOS activity that is already maximal after AT1R inhibition or (2) an absence of a cross talk between AT1Rs and AT2Rs in modulating NOS expression and/or activity.

In anaesthetized pigs, Solhaug et al. (1996b) observed that intrarenal pretreatment with the AT1R antagonist, A‐81988 also attenuated renal hemodynamic responses to intrarenal administration of l‐NAME in piglets aged ~3 weeks with a different response to that seen in adult pigs. Conversely, renal vasoconstrictor responses to ANG II were buffered by NO in a developmental pattern in isolated perfused kidneys removed from developing rabbits (Simeoni et al. 1997), suggesting an age‐dependent involvement of ANG II:NO interactions in the control of developing renal hemodynamics, at least in vitro. Therefore, it appears that early in life, there is a balance between ANG II and NO in influencing the renal vasculature, perhaps serving to protect it from any further increase in resistance that might occur with other constrictor factors in response to various harmful stimuli and thus help to preserve the essential filtration of the newborn kidney. These findings support an age‐dependent ANG II:NO interaction in the control of developing renal hemodynamic.

It is also possible that, in the present study in conscious lambs, the cardiovascular interactions between endogenously produced NO and ANG II (through ATR1s and/or AT2Rs) were influenced by other vasoactive factors such as endothelin‐1 (ET‐1) or prostaglandins (PGs). For example, we have provided evidence that endogenously produced PGs buffer the pressor and renal vasoconstrictor effects of ANG II in the postnatal period (Ebenezar et al. 2012), whereas there does not appear to be any role for endogenously produced NO in regulating these responses to exogenously administered ANG II (Ebenezar et al. 2012). In addition, NO (Smith et al. 2005) but not PGs (Ebenezar et al. 2010) buffer the hemodynamic effects of ET‐1 in the newborn period. Taken together, then, it appears that early in life, the primary vasodilators – PGs and NO – serve equal but opposite roles in regulating the primary vasoconstrictors –ANG II and ET‐1. To this, the present observations provide evidence that AT2Rs as well as AT1Rs appear to be involved in regulating the renal hemodynamic effects of endogenously produced NO.

There are several limitations to our study that should be considered. First, due to the time frame of experimental design determined by the postnatal interval over which ATRs expression is altered in sheep and the required clearance interval of the ATRs antagonists, evaluation of the hemodynamic effects of l‐NAME alone were not possible. Considerable experience with the model, however, and extensive testing of l‐NAME alone as well as ATR antagonists alone, have preceded this study, and allowed us to confidently interpret current findings in light of our previous studies. Second, there is limited information regarding the distribution and expression of both ATRs and NOS isoforms within the kidney of the developing sheep. This prevents us from further speculation into possible changes within the renal microvasculature that may have occurred under the treatments applied in our experiments. Finally, administration of ATR inhibitors and l‐NAME into the systemic circulation, prevent us from concluding whether the observed effects were related to central and/or peripheral mechanisms, and will warrant further investigation to more fully appreciate the underlying mechanisms of the ANG II:NO interactions.

In conclusion, the present study provides new insights into the balance between vasoconstrictor and vasodilator factors soon after birth. It appears that there is a complex interaction between the RAS and NO pathways in regulating cardiovascular homeostasis during the perinatal period. In fact, renal hemodynamic effects of NO in the developing lamb seem to be modulated by ATRs, which act to attenuate NOs vasodilator actions, with predominant effects in the newborn. Although other intrarenal vasoconstrictors may participate in the renal hemodynamic responses to systemic NO inhibition early in life, the RAS may function as one critical mechanism to counter regulate the profound vasodilator effects of NO in the newborn. Elucidating the physiological underpinnings of the role of NO and RAS in modulating the critical period of adaptation to extra uterine life could directly influence the management of the newborn at birth and may also impact on the development of diagnostic strategies and therapeutic approaches for the term and preterm infant and child in whom these two systems (NO and ANG II) are activated.

## Acknowledgments

Portion of this work was published in the proceedings of the Annual Meetings for Experimental Biology (Vinturache, A., Smith, F. G. Nitric oxide and renal haemodynamics during post natal maturation: Role of angiotensin receptors. *FASEB J*. 24: lb553, 2010). The authors gratefully acknowledge the excellent assistance provided for these experiments by Miss Lucy Yu and Dr. Wei Qi.

## Conflict of Interest

None declared.
